# Discovery of actionable genetic alterations with targeted panel sequencing in children with relapsed or refractory solid tumors

**DOI:** 10.1371/journal.pone.0224227

**Published:** 2019-11-20

**Authors:** Ji Won Lee, Nayoung K. D. Kim, Soo Hyun Lee, Hee Won Cho, Youngeun Ma, Hee Young Ju, Keon Hee Yoo, Ki Woong Sung, Hong Hoe Koo, Woong-Yang Park

**Affiliations:** 1 Department of Pediatrics, Samsung Medical Center, Sungkyunkwan University School of Medicine, Seoul, Republic of Korea; 2 Samsung Genome Institute, Samsung Medical Center, Sungkyunkwan University School of Medicine, Seoul, Republic of Korea; 3 Geninus Inc., Seoul, Korea; 4 Department of Translational Molecular Pathology, MD Anderson Cancer Center, University of Texas, Houston, Texas, United States of America; 5 Department of Health Science and Technology, Samsung Advanced Institute for Health Sciences and Technology, Sungkyunkwan University, Seoul, Republic of Korea; 6 Department of Molecular Cell Biology, Sungkyunkwan University School of Medicine, Seoul, Republic of Korea; 2nd Medical Faculty Charles University Prague and Faculty Hospital Motol, CZECH REPUBLIC

## Abstract

Advances in genomic technologies and the development of targeted therapeutics are making the use of precision medicine increasingly possible. In this study, we explored whether precision medicine can be applied for the management of refractory/relapsed pediatric solid tumors by discovering actionable alterations using targeted panel sequencing. Samples of refractory/relapsed pediatric solid tumors were tested using a targeted sequencing panel covering the exonic DNA sequences of 381 cancer genes and introns across 22 genes to detect clinically significant genomic aberrations in tumors. The molecular targets were tiered from 1 to 5 based on the presence of actionable genetic alterations, strength of supporting evidence, and drug availability in the Republic of Korea. From January 2016 to October 2018, 55 patients were enrolled. The median time from tissue acquisition to drug selection was 29 d (range 14–39), and tumor profiling was successful in 53 (96.4%) patients. A total of 27 actionable alterations in tiers 1–4 were detected in 20 patients (36.4%), and the majority of actionable alterations were copy number variations. The tiers of molecular alterations were tier 1 (clinical evidence) in 4 variants, tier 2 (preclinical evidence) in 8 variants, tier 3 (consensus opinion) in 2 variants, and tier 4 (actionable variants with a drug that is available in other countries but not in the Republic of Korea) in 9 variants. In one patient with relapsed neuroblastoma with *ALK* F1174L mutation and *ALK* amplification, lorlatinib was used in a compassionate use program, and it showed some efficacy. In conclusion, using a targeted sequencing panel to discover actionable alterations in relapsed/refractory pediatric solid tumors was practical and feasible.

## Introduction

The outcome of pediatric cancer has improved substantially over the past few decades; however, the prognosis of relapsed/refractory pediatric cancer remains poor, and a new approach is needed to improve the outcome. Recently, the tremendous progress in molecular biology has enhanced our understanding of tumorigenesis and cancer cell survival at the molecular level [1]. These advances have ultimately led to the development of targeted therapeutics, which directly inhibit the pathways responsible for tumorigenesis. Biomarker-driven targeted therapy has already been successfully implemented in clinical practice in adult oncology, such as in use of an epidermal growth factor receptor inhibitor to treat non-small cell lung cancer [2].

Recent advances in genomic technologies have improved the ability to detect diverse somatic and germline genomic aberrations in cancer patients. Together, the advances in genomic technology and targeted therapeutics are increasingly making possible the development of precision medicine, which can be applied regardless of cancer pathology. Several recent studies exploring the feasibility of a precision cancer medicine approach in pediatric oncology have been published [3–8], and these demonstrated that the application of clinical genomics was possible and that a substantial number of patients had actionable genetic alterations, indicating the potential for targeted therapy.

In this study, we explored the possibility of applying precision medicine to the management of refractory/relapsed pediatric solid tumors by discovering actionable alterations using targeted panel sequencing.

## Materials and methods

### Patients and sample collection

Patients with relapsed or refractory solid tumors, who were younger than 18 years at initial diagnosis, were considered eligible. Samples taken at the time of relapse were preferentially used for genomic analysis; however, stored samples taken at the time of diagnosis were used when samples at relapse could not be obtained. Both fresh frozen (FF) tissue and formalin-fixed, paraffin-embedded (FFPE) tissue samples were used. All tumor specimens were examined by a pathologist to determine the percentage of viable tumor cells in each sample and adequacy for sequencing.

This study was approved by the Institutional Review Board of Samsung Medical Center (IRB approval no. SMC 2015-11-053), and written informed consent was obtained from the participants and/or their parents or legal guardians.

### Targeted sequencing

A targeted sequencing panel (CancerSCAN^™^) to cover the exonic DNA sequences of 381 cancer genes and introns across 22 genes for rearrangement detection was used (S1 Table). This panel originally designed by the Samsung Genome Institute is available through company GENINUS. Tumor DNA (200 ng for FF or 300 ng for FFPE) was sheared in a Covaris S220 ultrasonicator (Covaris Inc., Woburn, MA, USA) and used for the construction of a library using CancerSCAN probes and an HSQ SureSelectXT reagent kit (Agilent Technologies Inc., Santa Clara, CA, USA) according to the manufacturers’ instructions. After the enriched exome libraries were multiplexed, the libraries were sequenced using the 100-bp paired-end mode of the TruSeq Rapid PE Cluster Kit and TruSeq Rapid SBS kit on the Illumina HiSeq 2500 sequencing platform (Illumina Inc., San Diego, CA, USA).

### Variant calling

DNA sequence data were aligned to the reference human genome (hg19) using the MEM algorithm in BWA version 0.7.5 [9]. Duplicate read removal was performed using Picard version 193 and SAMtools version 0.1.18 [10]. Local alignment was optimized using the Genome Analysis Toolkit (GATK) version 3.1–1 [11]. We also used BaseRecalibrator from GATK for base recalibration based on known single nucleotide polymorphisms and indels from Mills, Single Nucleotide Polymorphism Database Build 138 (dbSNP138), 1000G gold standard, 1000G phase1, and Omni 2.5.

Variant calling was only performed in regions targeted in CancerSCAN^™^ version 2. We detected single nucleotide variants using two tools: MuTect version 1.1.4 and LoFreq version 0.6.1 [12,13]. Falsely detected variants were filtered out from abnormally aligned strand-biased and clustered reads using scripts developed in-house. ANNOVAR was used to annotate the detected variants with various resources, including dbSNP138, Catalogue of Somatic Mutations in Cancer (COSMIC), the Cancer Genome Atlas (TCGA), Exome Sequencing Project (EPS5400), 1000 Genomes Project, Exome Aggregation Consortium database (ExAC03), and the in-house Korean SNP database. Indels were detected with Pindel version 0.2.4 [14] and annotated using ANNOVAR. To filter out germline variants, we applied two algorithms: (1) except for hotspot mutations, variants with an allele frequency ≥97% were filtered out and (2) suspect germline variants were filtered out based on whether the allele frequency was ≥1% of the previously mentioned database or ≥3% of 480 samples from healthy Korean subjects. We used CancerSCAN^™^ software [15] to detect copy number variations (CNVs). In CancerSCAN^™^, the software “Depth of Coverage” in GATK version 3.1–1 was used to calculate sequencing coverage in each exon. The median of the mean coverage for the total number of exons was calculated and divided by the gained average for the normalization of the mean coverage of exons. The median value of normalized exons of pattern matched normal reference datasets (HapMap cell lines and FFPE tissues from normal patients) was used as a reference value. The normalized median value of exons for each patient was subsequently divided by the reference value and transformed to the binary logarithm. Tumor purity for adjusting CNVs was calculated using normalized coverage and B allele frequencies at all exons. Using normal samples, coverage of the sample was fitted, and subsequently, major ploidies and copy numbers in the neutral region were determined. Based on the inferred copy neutral region and major ploidies, tumor purity was calculated using the relationship between B allele frequencies and tumor ploidy data. Finally, we defined “copy number deletion” as a copy number of less than 0.7, and “copy number amplification” as a copy number of more than 4 using the above method.

### Tier classification and selection of potential targeted agents

Identified molecular alterations were assessed using a multidisciplinary panel regarding biological relevance and potential druggability. Clinical or preclinical evidence was collected using ClinVar (https://www.ncbi.nlm.nih.gov/clinvar), My Cancer Genome (https://www.mycancergenome.org), Targeted Therapy Database (http://www.mmmp.org), JAX-Clinical Knowledgebase (https://ckb.jax.org/), and the U.S. National Institute of Health clinical database (https://clinicaltrials.gov/). Variants and drug interactions were also manually reviewed in the literature. The molecular targets were tiered from 1 to 5: 1a, clinical evidence of the same alteration in the same disease; 1b, clinical evidence of the same alteration in a different disease; 2a, preclinical evidence of the same alteration in the same disease; 2b, preclinical evidence of the same alteration in a different disease; 3, consensus opinion; 4, actionable variants with drugs that are available in other countries for use in clinical trials or FDA-approved drugs for the same or other diseases but that are not available in Korea; and 5, no actionable genetic variants.

## Results

### Patients and specimens

Fifty-five patients were enrolled between January 2016 and October 2018. The characteristics of the patients and specimens are summarized in Table 1. The most frequent disease was neuroblastoma (n = 16, 29.1%). For 41 patients, tissue samples taken at recurrence were analyzed. For the remaining patients (n = 14), primary tumors were sequenced because of tissue unavailability at relapse. For 2 patients, sequencing could not be performed because of poor DNA quality. The median time from tissue procurement to a variant calling-based clinical decision was 29 d (range: 14 to 39), including 7 d (range: 2 to 15) for DNA preparation, 19 d (range: 7 to 29) for sequencing and bioinformatics analysis, and 2 d (range: 0 to 5) for deciding on the targeted agents.

**Table 1:** Patients and specimens characteristics value.

Age at enrollment, median (range, years)	10.7 (0.8–20.7)
Sex, No. (%)	
Male	30 (54.5)
Female	25 (45.5)
Diagnosis, No. (%)	
Neuroblastoma	16 (29.1)
Rhabdomyosarcoma	9 (16.4)
Medulloblastoma	5 (9.1)
High-grade gliomas	4 (7.3)
Ewing sarcoma	3 (5.5)
Hepatoblastoma	3 (5.5)
Osteosarcoma	3 (5.5)
Alveolar soft part sarcoma	2 (3.6)
Wilms tumor	2 (3.6)
Others^a^	8 (14.5)
**Tumor samples for analysis**	
Initial diagnosis	14 (25.5)
Relapse/refractory/progression	41 (74.5)
**Type of sample**	
Paraffin-embedded tissue	34 (61.8)
Fresh-frozen tissue	21 (38.2)

^a^Others include angiosarcoma, CNS embryonal tumor, desmoplastic small round cell tumor, EBV-associated lymphoepithelioid carcinoma, epithelioid sarcoma, inflammatory myofibroblastic tumor, malignant rhabdoid tumor, and yolk sac tumor.

### Results of targeted sequencing and tier classification

**Fig 1:**
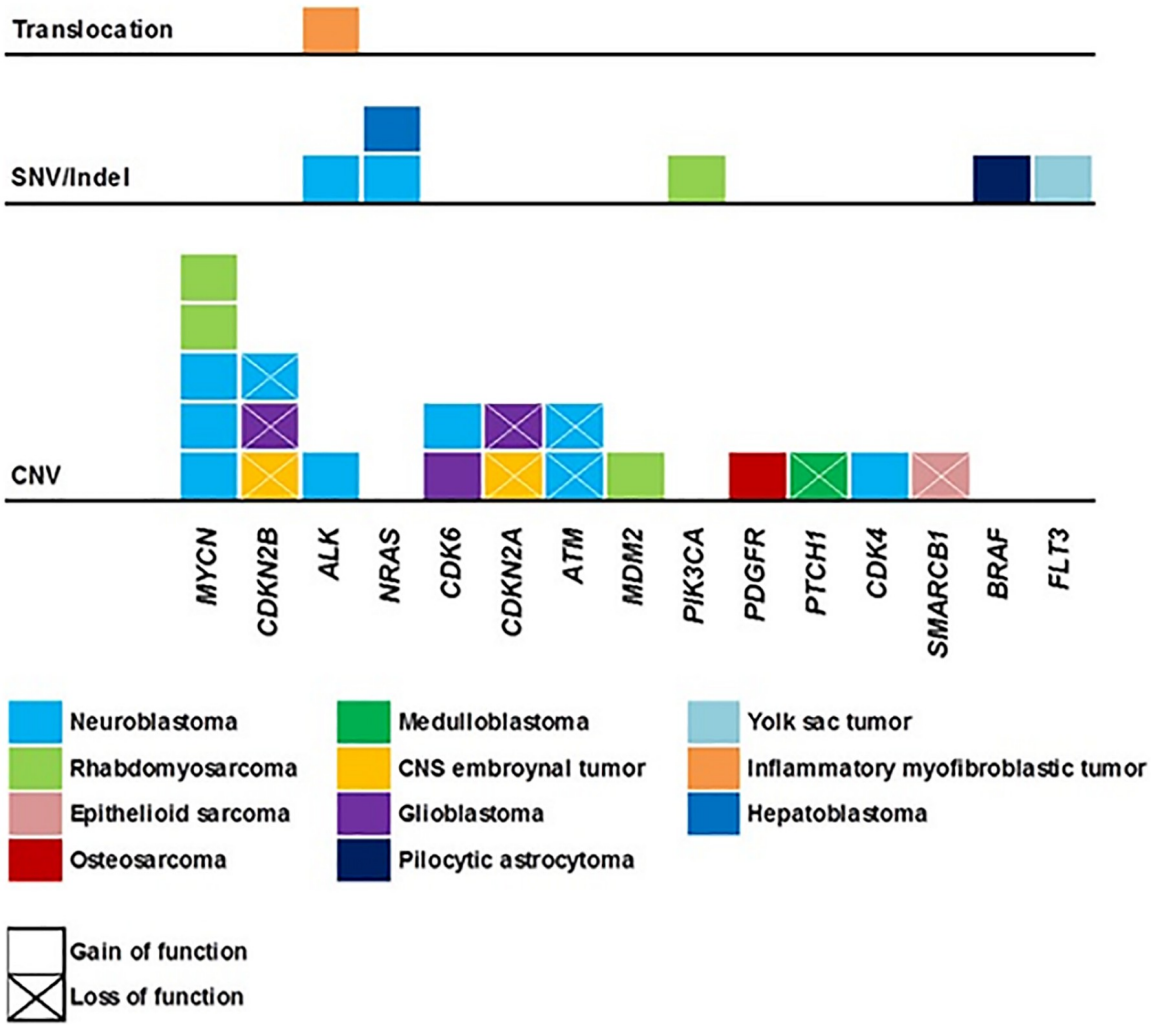
Summary of actionable alterations. A total of 27 actionable alterations were detected in 20 patients (35.1%), and the majority of actionable alterations were copy number variations.

Detected genetic variants were illustrated in S2 Table. The average target depth for all samples was 874.53× (SD = 234.03), and the median tumor mutation burden was 14.1 (range 0–43.7) mutations/Mb. A total of 27 actionable alterations were detected in 20 patients (36.4%), and the majority of actionable alterations were CNVs (Fig 1). In a patient with an inflammatory myofibroblastic tumor, conventional fluorescence in situ hybridization (FISH) analysis showed negative *ALK* rearrangement, but a *TPM4*-*ALK* translocation was detected by targeted sequencing.

Molecular alterations were tiered from 1 to 5; 3 variants were classified into tier 1, 8 variants were classified into tier 2, 2 variants were classified into tier 3, 10 variants were classified into tier 4, and the other 37 patients were classified into tier 5 with no actionable alteration. For the alterations in tiers 1–4, potential matched targeted agents were selected for each variant. The details of the genetic variants in tiers 1–4 are summarized in Table 2 along with the references used in the tier classification [16–30].

**Table 2:** Actionable alterations and tier classification.

No.	Diagnosis	Actionable alterations	Tier	Potential targeted agent	References
002	Rhabdomyosarcoma	*MYCN* amplification	4	BET bromodomain inhibitor	[16]
003	Neuroblastoma	*NRAS* Q61K, *CDK4* amplification, *MYCN* amplification	2a 4 4	Everolimus Palbociclib BET bromodomain inhibitor	[17] [18,19] [16]
004	Rhabdomyosarcoma	*MDM2* amplification	4	Selinexor	[20]
007	Rhabdomyosarcoma	*MYCN* amplification	4	BET bromodomain inhibitor	[16]
009	Rhabdomyosarcoma	*PIK3CA* H1047R	4	PI3K/AKT/mTOR pathway inhibitor	[21]
010	Osteosarcoma	*PDGFRA* amplification	3	Pazopanib	[22,23]
017	Medulloblastoma	*PTCH1* deletion	4	Vismodegib	[24]
023	Neuroblastoma	*MYCN* amplification	4	BET bromodomain inhibitor	[16]
024	Glioblastoma multiforme	*CDK6* amplification	4	Palbociclib	[18,19]
030	Epithelioid sarcoma	*SMARCB1* deletion	4	Tazemetostat	NCT03213665
031	Neuroblastoma	*CDK6* amplification	2b	Palbociclib	[18,19]
034	Neuroblastoma	*ATM* deletion, *CDKN2B* deletion	1b 2b	Olaparib Palbociclib	[25] [18,19]
037	Hepatoblastoma	*NRAS* Q61R	2b	Everolimus	[17]
038	Glioblastoma multiforme	*CDKN2A/B* deletion	2a	Palbociclib	[18,19]
042	Neuroblastoma	*ATM* deletion	1b	Olaparib	[25]
046	Pilocytic astrocytoma	*BRAF* L485F	3	Vemurafenib	[26,27]
052	Yolk sac tumor	*FLT3* T820N	2b	Sorafenib	[28]
059	CNS embryonal tumor	*CDKN2A/B* deletion	2b	Palbociclib	[18,19]
060	Neuroblastoma	*ALK* F1174L, *ALK* amplification	2a	Lorlatinib	[29]
064	Inflammatory myofibroblastic tumor	*ALK* translocation	1a	Crizotinib	[30]

### Example of clinical application

**Fig 2:**
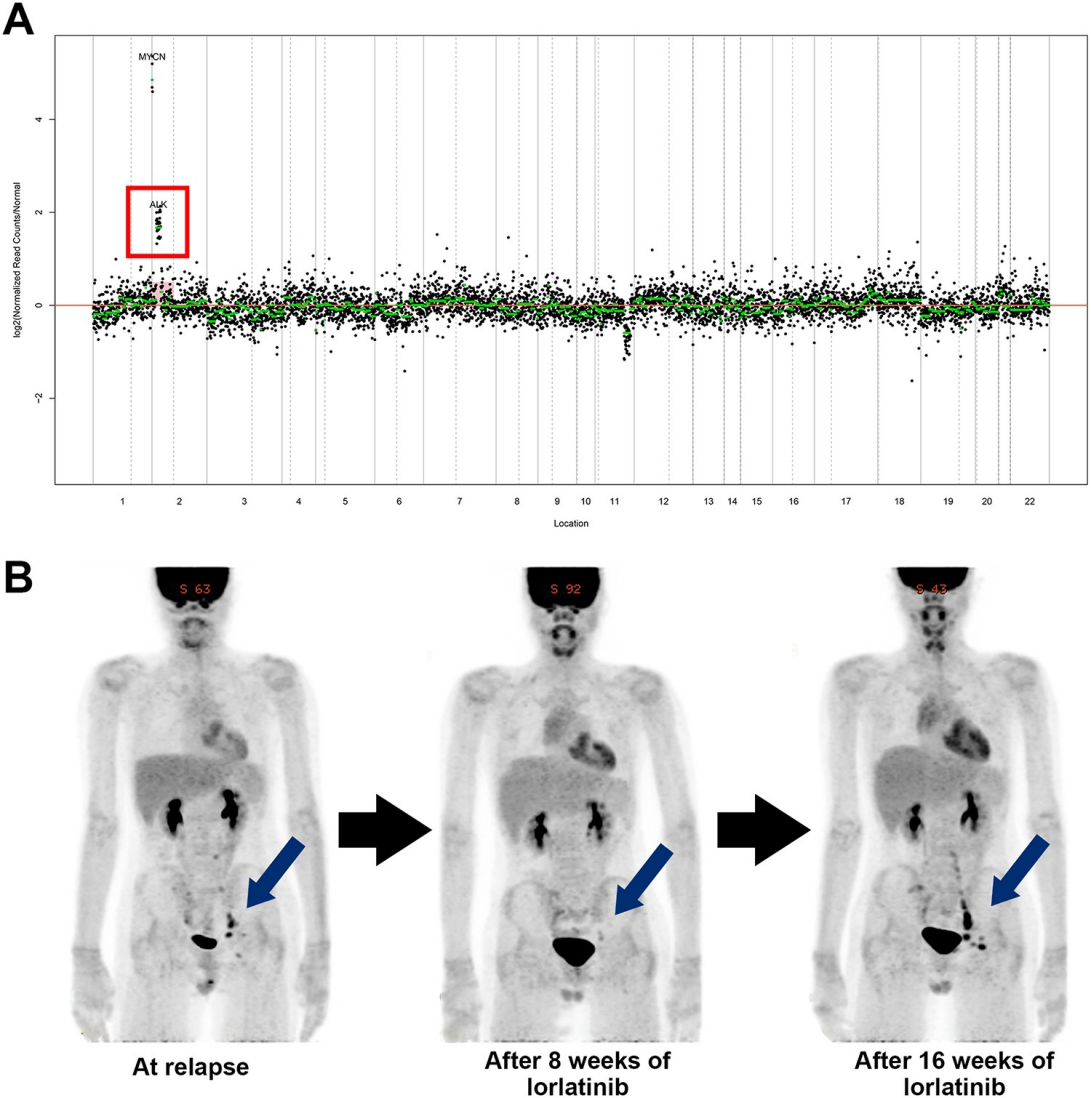
An example of clinical application. (A) In a patient with relapsed neuroblastoma, an *ALK* F1174L mutation and *ALK* amplification were both detected. (B) Eight weeks after the use of lorlatinib, the tumor responded considerably but showed subsequent relapse after 16 weeks of treatment.

As an example of clinical application, a patient (No. 060) was diagnosed with stage 4, *MYCN* amplified neuroblastoma. He underwent induction chemotherapy, surgery, and tandem high-dose chemotherapy, but he relapsed immediately after the second high-dose chemotherapy. Our analysis of the relapsed tumor tissue sample revealed an *ALK* F1174L mutation and together with an *ALK* amplification (Fig 2A). Crizotinib, a 1^st^ generation *ALK* inhibitor, has been reported to have potency in in vitro studies with neuroblastoma cell lines [31], and it has been tested in a Phase I clinical trial for the treatment of pediatric solid tumors [32]. However, certain mutations such as *ALK* F1174L are known to have intrinsic crizotinib resistance, and Infarinato NR et al. demonstrated that lorlatinib (PF-06463922; Pfizer Oncology, Groton, CT, USA) had the potential to overcome crizotinib resistance against F1174L *ALK*-mutated xenograft tumors [29]. With this background, a clinical trial with lorlatinib in *ALK*-driven neuroblastoma is now being conducted by New Approaches to Neuroblastoma Therapy (NANT) Consortium (NCT03107988). Based on these facts, lorlatinib was used in a compassionate use program for this patient. After 8 weeks of treatment, the size of several metastatic lymph nodes along the left iliac chain decreased on computed tomography (CT) scan (largest size 1.6 cm → 1.0 cm). Positron emission tomography (PET) and metaiodobenzylguanidine (MIBG) scan also showed response (maximal SUV 6.6 → 2.4 and MIBG score 2 → 0). However, the tumor relapsed again after 16 weeks of treatment (Fig 2B).

## Discussion

In this study, the possibility of the use of precision medicine for the management of refractory/relapsed pediatric solid tumors was assessed through a prospective study using a targeted sequencing panel. Following the advances in genomic technology and the development of targeted therapeutics, several studies have explored the feasibility of precision medicine in pediatric oncology [3–8]. In these studies, various genomic technologies were utilized, including targeted sequencing, array comparative genomic hybridization (aCGH) to detect copy number variation, whole exome sequencing, and RNA sequencing. Genetic alterations affecting clinical decisions were reported in 31 to 73% of patients, depending on the study. The differences in the frequency of alterations are thought to be due to the various technologies used as well as the definition of actionable alterations used by each group. In our study, we used a targeted sequencing panel. Targeted panel sequencing has many advantages, including the relative simplicity of the method to detect known variants with high coverage and low complexity [33], thus making it easy to apply in clinical practice. With the targeted sequencing panel, tumor profiling was possible in 96.4% of patients, and the turnaround time of a median 29 d was acceptable for a clinical trial.

In our study, actionable alterations were found in 20 (36.4%) patients, and CNVs were the most frequent actionable targets. These results are consistent with the results of the Individualized Cancer Therapy (iCat) study by Harris MH, in which the most common actionable alteration leading to an iCat recommendation was a copy number alteration [4]. Somatic mutation frequency in pediatric cancer is much less than that of adult cancer; Lawrence MS *et al* showed that median frequency of non-synonymous mutations varied by more than 1,000-fold across cancer types, and pediatric cancers showed frequencies as low as 0.1/Mb [34]. Another study showed that tumor mutational burden increases significantly with age, showing a 2.4-fold difference between age 10 and age 90 years [35]. In a recently published article that studied the landscape of genomic alterations across multiple childhood cancers, the mutation frequency and significantly mutated genes in pediatric cancer were markedly different compared to those reported previously for adult cancers [36]. These findings suggest that the genomic profiles of childhood cancer are different from those of adult tumors, so the approach to pediatric cancers needs to be different from that for adult cancers. The targeted sequencing panel used in this study was originally designed for adult cancers, so a panel designed specifically for pediatric cancer will be needed. The pediatric panel needs to include not only actionable genes but also genes used as diagnostic or prognostic markers for pediatric cancers. Moreover, the information obtained from targeted sequencing only provides a limited understanding of tumor biology, and further comprehensive research, including transcriptome, proteomic, and epigenomic analyses, will be needed to improve the detection of potential actionable alterations. In vitro drug screening with patient-derived cells represents another possible option to screen for potential drugs for use in precision medicine.

One of the clinical limitations of these approaches is that drug availability is limited even though actionable alterations were found. This is an inevitable problem in the pediatric population, especially in the Republic of Korea. Although considerable progress has been made, the development of drugs for childhood cancer is still in its early stages. Recently, the Innovative Therapies for Children with Cancer (ITCC) Consortium suggested a new adaptive design for early phase clinical trials in children and adolescents to facilitate drug development for childhood and adolescent cancers [37]. In parallel with these efforts, accessible clinical trials need to be expanded globally in order to increase the therapeutic options available to children and adolescents with relapsed or refractory disease.

We preferred samples taken at the time of relapse because there can be new additional mutations and clonal evolution at relapse. Some of patients in this study had samples taken at both diagnosis and relapse, and genomic changes in these patients were studied in our previous work [38]. In the previous study, 72.6% of SNVs in primary tumors were found in recurrent lesions, however, 27.2% of SNVs had newly occurred in recurrent tumors [38].

There are several limitations in our study. First of all, germline samples were not analyzed together with tumor samples, so interpretation of germline variants was limited in this study. Also, there are also limitations in interpreting variants of unknown significance detected, and more in-depth analysis including *in silico* analysis will be necessary in the future.

In conclusion, using a targeted sequencing panel to discover actionable alterations in relapsed/refractory pediatric tumors was practical and feasible. More intensive genomic studies and efforts to increase access to potential drugs are needed to improve the application of precision medicine in pediatric cancer.

## Supporting information

S1 Table List of target genes. Target genes included in targeted sequencing panel (CancerSCAN^™^) were listed.
(XLSX)

S2 Table Genetic variants identified were listed. (XLSX)

S1 Data Data set. (XLSX)
